# The N-Terminal Domain of the Arenavirus L Protein Is an RNA Endonuclease Essential in mRNA Transcription

**DOI:** 10.1371/journal.ppat.1001038

**Published:** 2010-09-16

**Authors:** Benjamin Morin, Bruno Coutard, Michaela Lelke, François Ferron, Romy Kerber, Saïd Jamal, Antoine Frangeul, Cécile Baronti, Rémi Charrel, Xavier de Lamballerie, Clemens Vonrhein, Julien Lescar, Gérard Bricogne, Stephan Günther, Bruno Canard

**Affiliations:** 1 Architecture et Fonction des Macromolécules Biologiques, CNRS and Universités d'Aix-Marseille I et II, UMR 6098, ESIL Case 925, Marseille, France; 2 Department of Virology, Bernhard-Nocht-Institute for Tropical Medicine, Hamburg, Germany; 3 Unité des Virus Emergents UMR190, Université de la Méditerranée & Institut de Recherche pour le Développement, Marseille, France; 4 Global Phasing Ltd., Cambridge, United Kingdom; 5 School of Biological Sciences, Nanyang Technological University, Singapore, Singapore; University of California Irvine, United States of America

## Abstract

*Arenaviridae* synthesize viral mRNAs using short capped primers presumably acquired from cellular transcripts by a ‘cap-snatching’ mechanism. Here, we report the crystal structure and functional characterization of the N-terminal 196 residues (NL1) of the L protein from the prototypic arenavirus: lymphocytic choriomeningitis virus. The NL1 domain is able to bind and cleave RNA. The 2.13 Å resolution crystal structure of NL1 reveals a type II endonuclease α/β architecture similar to the N-terminal end of the influenza virus PA protein. Superimposition of both structures, mutagenesis and reverse genetics studies reveal a unique spatial arrangement of key active site residues related to the PD…(D/E)XK type II endonuclease signature sequence. We show that this endonuclease domain is conserved and active across the virus families *Arenaviridae*, *Bunyaviridae* and *Orthomyxoviridae* and propose that the arenavirus NL1 domain is the *Arenaviridae* cap-snatching endonuclease.

## Author Summary

The *Arenaviridae* virus family includes several life-threatening human pathogens that cause meningitis or hemorrhagic fever. These RNA viruses replicate and transcribe their genome using an RNA synthesis machinery for which no structural data currently exist. They synthesize viral mRNAs using short capped primers presumably acquired from cellular transcripts by a ‘cap-snatching’ mechanism thought to involve the large L protein, which carries RNA-dependent RNA polymerase signature sequences. Here, we report the crystal structure and functional characterization of an isolated N-terminal domain of the L protein (NL1) from the prototypic arenavirus: lymphocytic choriomeningitis virus. The NL1 domain is able to bind and cleave RNA. The 2.13 Å resolution crystal structure of NL1 reveals a type II endonuclease α/β architecture similar to the N-terminal end of the influenza virus PA protein. Superimposition of both structures and mutagenesis studies reveal a unique spatial arrangement of key active site residues related to the PD…(D/E)XK type II endonuclease signature sequence. Reverse genetic studies show that mutation of active site residues selectively abolish transcription, not replication. We show that this endonuclease domain is conserved and active across the virus families: *Arenaviridae*, *Bunyaviridae* and *Orthomyxoviridae* and propose that the arenavirus NL1 domain is the *Arenaviridae* cap-snatching endonuclease.

## Introduction

The *Arenaviridae* family includes 22 viral species into a single genus Arenavirus, with new species awaiting classification [1], [2]. They cause chronic and asymptomatic infections in rodents, and occasional transmission to man may result in life-threatening meningitis and/or hemorrhagic fever. *Lymphocytic choriomeningitis virus* (LCMV) is the prototypic species and first arenavirus isolated in 1933. Because its natural host is the common house mouse (*Mus musculus*), LCMV is the only known arenavirus presumably exhibiting a worldwide distribution. LCMV is a human pathogen of significant clinical relevance, causing central nervous system disease, congenital malformation, choriomeningitis, and systemic and highly fatal infection in immuno-compromised, organt transplant recipient patients [3], [4], [5], [6]. Humans are generally infected through the respiratory tract after exposure to aerosols, or by direct contact with infectious material.

Arenaviruses are enveloped viruses with a bisegmented negative single-strand RNA genome. Each RNA segment, called large (L; ∼7.2 kb) and short (S; ∼3.5 kb), contains two open reading frames in mutually opposite orientations and use an ambisense coding strategy to direct the synthesis of two polypeptides [7]. Between the two open reading frames of each segment resides a non-coding intergenic region (IGR), composed of a sequence predicted to form a stable hairpin structure [8]. The S RNA encodes the viral nucleoprotein (NP; ∼63 kDa) and glycoprotein precursor (GPC; ∼75 kDa), whereas the L RNA encodes a small RING finger protein (Z; ∼11 kDa) and a large protein (L; ∼250 kDa) which is the viral RNA-dependent RNA polymerase (RdRp). The two RNA genomes are encapsidated by the NP, which is the most abundant protein in virions and infected cells, and act as templates for two fundamentally different processes, RNA replication and transcription. During RNA replication, the L protein first binds to the 3′-end of RNA templates and reads them from end to end to direct the synthesis of encapsidated full-length anti-genomes. During transcription, the RdRp stops RNA synthesis at a pause site located near the IGR [7]. The newly synthesized mRNA molecules have a non-polyadenylated 3′-end with a heterogeneous sequence mapped within the predicted hairpin in the IGR [9]. Furthermore, non template-directed sequences have been identified at the 5′-end of the subgenomic mRNA [10]. These sequences are variable in length [9], [10], [11] and terminate with a 5′-cap structure, which suggests the presence of a cap-snatching mechanism for arenaviruses. In this process, originally described for influenza viruses [12], [13] and bunyaviruses [14], the viral RdRp binds cellular mRNAs caps and ‘steals’ them using an endonuclease activity, located in the influenza PA subunit [15], [16], and presumably in L protein of bunyaviruses. These short capped RNAs are then used as primers for mRNA synthesis. The arenavirus L protein is an essential element in genome replication and transcription [17]. It is the largest viral protein composed of approximately 2200 amino-acid (aa) residues, and sequence analysis using homologous proteins led to the prediction of several conserved domains [18], [19]. A biological function can be inferred for the L3 domain containing conserved and typical RdRp signature sequence motifs [19], [20]. For Tacaribe virus, both domains L1 and L3 interact with the Z protein [21]. By analogy with influenza and bunyaviruses, the L protein may also carry activities and domains responsible for a cap-snatching mechanism that would account for the sequence diversity found at the 5′-end of RNA transcripts. The expression and purification of such a large viral polymerase is problematic and has not been documented.

We report here the first crystal structure of an *Arenaviridae* L protein domain at 2.13 Å resolution, that of the N-terminus domain of the LCMV L protein. We show that this domain is able to bind nucleotides, with a preference for UTP, and RNA. Structural comparison with the N-terminal part of the influenza virus PA protein characterizes unambiguously the domain as an endonuclease. Sequence and secondary structure analysis of L proteins from various *Bunyaviridae* family members predict that their N-terminal end carries a similar endonuclease activity, that we demonstrate for Toscana virus (TOSV) (genus *Phlebovirus,* family *Bunyaviridae*). Activity assays and mutagenesis show that the arenavirus endonuclease exhibits sequence-specificity with a preference for uracil-containing substrates. Lastly, reverse genetics studies correlate expression of endonuclease activity with the selective production of mRNA, making the N-terminus domain of the L protein a likely candidate to be involved in the cap-snatching mechanism of arenaviruses.

## Results

### Delineation of an Arenavirus L Protein Domain and its Crystal Structure

Based on aa sequence conservation across arenaviruses and on the presence of a potential nucleotide-binding site, we designed cDNA constructs encoding aa residues 1 to ∼250 for the N-terminal end of four arenavirus (Pirital virus (PIRV), Lassa fever virus (LASV), Parana virus (PARV), and LCMV) L proteins. All four domains were expressed as soluble recombinant proteins. We observed a self-limited proteolysis of the Parana arenavirus N-terminus L domain which prompted us to refine boundaries into a shorter 196 aa form, hereafter named “NL1”, fully included in the previously predicted arenavirus L1 domain (1–250 aa) [19]. The construct was expressed in *E.coli* and purified, but yielded crystals diffracting to 8 Å. However, the homologous 196 residues domain of LCMV yielded well-diffracting crystals. The atomic structure of NL1 was first determined by the SAD technique with seleno-methionylated crystals that diffracted to 3.4 Å. The structure was refined using a native data set at 2.13 Å resolution (Table 1). Two NL1 molecules are present within the asymmetric unit. Residues 1–191 are visible for one molecule whilst only 1–175 could be modelled for the other NL1 molecule owing to high mobility of the C-terminal end of helix α7.

**Table 1:** Data Collection and Refinement Statistics.

Data collection and refinement statistics	LCMV NL1
Space group	C222_1_
Cell dimensions	
*a*, *b*, *c* (Å)	145.0, 159.3, 52.6
Resolution (Å)	107.25−2.13 (2.19–2.13)*
Measured/unique reflections	231,821/33,999
*R*_merge_	3.9 (45.2)
*<I*/σ*I>*	27.1 (1.9)
Completeness (%)	97.9 (97.8)
Redundancy	6.8 (2.9)
Resolution (Å)	72.50−2.13
No. reflections	33,931
*R*_work_/*R*_free_(%)	19.86/22.21
No. atoms	3,297
Protein	3,011
Water	253
*B*-factors	
Protein	57.6
Water	68.2
R.m.s. deviations	
Bond lengths (Å)	0.010
Bond angles (°)	1.14

*Values in parentheses are for the highest-resolution shell.

### The LCMV NL1 Domain Exhibits a Type II Endonuclease Fold

**Figure 1:**
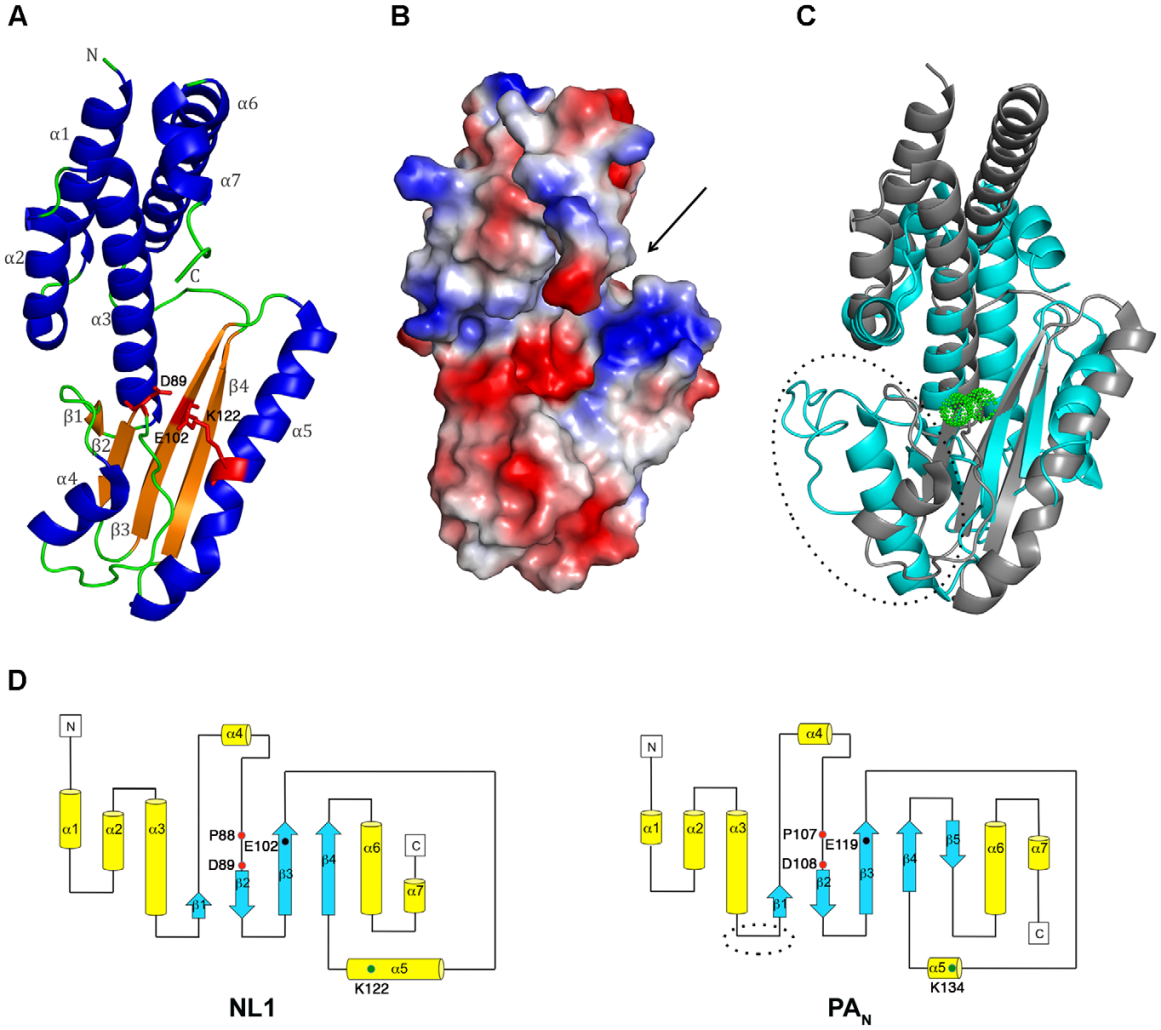
NL1 structure and comparisons with the influenza PA_N_ structure. **A**, Cartoon-representation of the NL1 structure. Secondary-structure elements are labelled and colored as follows: α-helices, blue, β-strands, orange, and loops, green. Side-chains for residues within the NL1 endonuclease active site are shown as red sticks and labelled. **B**, Electrostatic surface representation showing NL1 in the same orientation as in panel (**A**). The arrow indicates the putative RNA binding groove and the active site crevice. Negative charges are in red and positive charges in blue and neutral in white. **C**, Superimposition (view in the same orientation as in **A**) of the structures of NL1 (grey) and PA_N_ (PDB code:2W69, cyan) highlighting their shared structural core as well as variations in the form of an extra loop only present in the PA_N_ structure (circled). The two Mn^2+^ ions in the PA_N_ structure active site are depicted as green spheres. **D**, Topology diagrams of the NL1 (left) and PA_N_ (right) structures. α-helices are represented as yellow tubes and β-strands are blue arrows. The extra-loop of PA_N_ protein is circled as in panel **C**. Key residues from the endonuclease active site (PD, E/D, and K), are schematically depicted by colored dots and labelled, highlighting the fact that they project from conserved structural elements between the influenza PA_N_ protein and the arenavirus NL1 domain.

The LCMV NL1 monomer structure has approximate dimensions of 59 Å ×37 Å ×27 Å. It features four mixed β-strands forming a twisted plane surrounded by seven α-helices (Figure 1A). The two anti-parallel strands β1 and β2 are connected by helix α4, whereas the two parallel strands β3 and β4 are connected by the long helix α5. These two helices run parallel to the central β-sheet and are disposed at the same side of the latter. On the opposite side of the β-sheet, helix α3 is surrounded at its extremity by two N-terminal (α1 and α2) and C-terminal helices (α6 and α7). A search for similar protein folds using the DALI server [22] returned the PA N-terminal domain structure that was recently identified as a type II endonuclease domain [15], [16]. The structural match with published molecular structures of the influenza PA N-terminal domains (PA_N_) returns a Z-score of 5.7 and an r.s.m.d. of 3.9 Å for 121 superposed aa (PDB code 3EBJ) and Z-score 5.2, r.m.s.d. 4 Å for 122 aa (PDB code 2W69). As was the case for PA_N_, other type II endonuclease proteins are also recovered: the Tt1808 hypothetical protein from *Thermus Thermophilus* HB88 (PDB code 1WDJ, Z-score 3.8, r.m.s.d. 3.4 Å for 81 aa), and the restriction endonuclease *SdaI* (PDB code 2IXS, Z-score 3.6, r.m.s.d. 6.3 Å for 104 aa).

**Figure 2:**
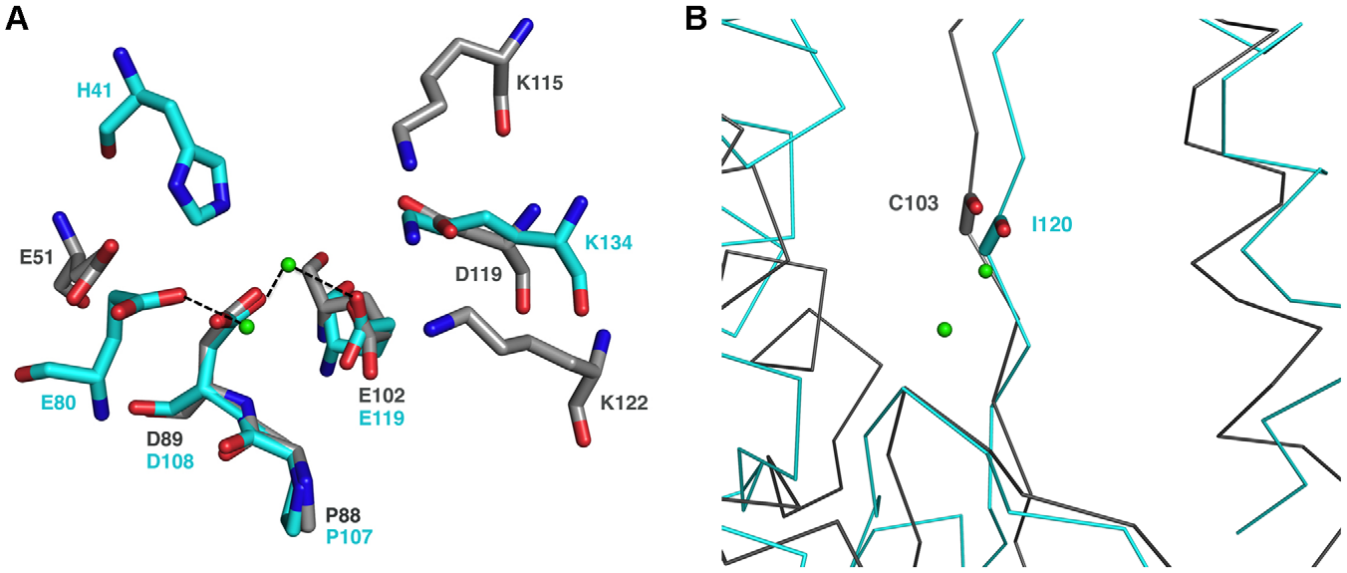
The endonuclease active site. **A**, Structure-based superimposition of the endonuclease active site from the influenza PA_N_ protein and the arenavirus NL1 domain. Putative active site residues of NL1 are shown as grey sticks and the active site of PA_N_ (PDB code 2W69) in cyan. The two Mn^2+^ ions present in the PA_N_ structure (but not in the present NL1 domain crystal structure) are shown as light green spheres with their closest ligand indicated by a dashed line. **B**, C-alpha trace ribbon-representation of the superimposition of the endonuclease active site from the influenza PA_N_ (cyan) protein and the arenavirus NL1 domain (grey). The carbonyl main-chain of PA_N_ I120 and NL1 C103 are shown in sticks. The metal ions are shown as light green spheres.

The β-sheet forms a negatively charged cavity creating a binding site for divalent cations, whilst above that cavity, the C-terminal end of helix α5 forms a positively charged patch and a concave surface that is likely to accommodate the RNA substrate (Figure 1B, arrow). The PA protein constitutes one subunit that associates with PB1 and PB2 to form the heterotrimeric influenza virus polymerase. Its N-terminal domain PA_N_ hosts the RNA cap-snatching endonuclease activity [15], [16]. Both NL1 and PA_N_ share a similar core structure. Except for the absence of a fifth β-strand in NL1, all other secondary structure elements are conserved (Figure 1C) and the overall topology of these two structures is very similar (Figure 1D), albeit with interesting differences in the vicinity of the PA_N_ active site (discussed below). At the aa sequence level, NL1 shares the conserved active site sequence motif characteristic of type II endonucleases: PD…(D/E)XK. In NL1, the corresponding residues are P88, D89…E102, and either K115 or K122 (Figure S1A, B). The identity of the distal lysine is not certain since it is found at different positions in the primary sequence, as is the case for influenza virus. The influenza PA_N_ domain was crystallized either in the presence of magnesium or manganese ions in the active site which comprises five conserved catalytic residues: H41, E80, D108, E119 and K134. A structural superimposition of the arenavirus NL1 and influenza PA_N_ active sites shows that the side-chains of three evolutionary-conserved residues within arenaviruses (P88, D89 and E102) closely superimpose with P107, D108 and E119 of the influenza virus PA_N_ protein, pointing to a common function for these residues (Figure 2A and Figure S1B). Upon superimposition with PA_N_, one Mn^2+^ ion needed for the enzymatic reaction coordinated by D108 in the PA_N_ active site, falls at right distances to be coordinated by the carboxylate side-chains of D89 and E102. NL1 was crystallized without metal ions and a water molecule is found close to the position that should be occupied by the divalent metal. Interestingly, no close structural match is found neither for H41 nor K134 of the influenza virus PA_N_. This points to differences between the two active sites since His41 was proposed to play a catalytic role in the influenza PA_N_. However, we note that another possible contributor could be NL1 C103 main-chain carbonyl as it superimposes quite well with PA_N_ I120 main-chain carbonyl (Figure 2B). The triad made of K115, D119, and K122 in NL1 is spatially equivalent to K134 in PA_N_. In summary, despite no aa sequence homology, the active site structures of the influenza PA_N_ and LCMV NL1 domains are clearly related but not identical (Figure 1C, 2), strongly suggesting that these two domains exhibit closely related enzymatic activities (see below).

### The NL1 Endonuclease Fold is Conserved Amongst *Bunyaviridae*

In addition to *Arenaviridae* and *Orthomyxoviridae*, *Bunyaviridae* is the other family of virus to possess a segmented negative-strand RNA genome. It contains four genera of animal viruses (*Orthobunyavirus, Phlebovirus, Nairovirus, Hantavirus*) and one genus of plant virus (*Tospovirus*) [23]. Although the genomic organisation differs between these three virus families, *Bunyaviridae* are also thought to use a cap-snatching mechanism to prime mRNA synthesis [24]. Arenaviruses, and *Bunyaviridae* share a conserved RdRp motif within their large L protein, as well as a conserved N-terminus domain [18]. Amino-acid sequence alignments, assisted by secondary structured prediction, of the N-terminal part of LCMV and *Bunyaviridae* L protein reveal that the latter also possesses the conserved active site motifs characteristic of type II endonucleases (Figure S2A). However, we could identify the catalytic motif within the L protein N-terminal end for only four out of the five bunyavirus genera: *Orthobunyavirus*, *Phlebovirus*, *Hantavirus* and *Tospovirus*. The L protein of *Nairovirus* is much larger (∼4000 aa) than the L protein of other members of the *Bunyaviridae* family (∼2200 aa). The putative endonuclease catalytic motif was located after aa ∼700, the N-terminal of *Nairovirus* L protein being assigned as a so-called OTU-like domain [25].

Secondary structure predictions were used to draw the topology diagram of the NL1-like domain for each genera (Figure S2B). As expected from the sequence alignment, each genus seems to share a β-sheet with a variable number of β-strands. Furthermore, the PD catalytic motifs are in each case located in a loop before a β-strand, as expected. The PUMV, HLCV and RVFV NL1-like domains are more closely related to LCMV NL1 than are the TOMV and CCGV. The TOMV NL1-like domain contains 6 β-strands and shares the PD motif just upstream the first β-strand, whereas it is just upstream the second β-strand in the case of NL1 and PA_N_. Finally, the structural organization of the putative CCGV endonuclease domain seems to diverge even further from the others. Indeed, whereas the conserved lysine is shared by the same helix for all the domains, that of *Nairovirus* may be located at the end of the β4 strand (Figure S2B). Thus we conclude that the endonuclease motif is conserved across four animal virus genera *Orthobunyavirus*, *Phlebovirus*, *Nairovirus* and *Hantavirus.*

### NL1 is a Mn^2+^-Dependent RNA Endonuclease

**Figure 3:**
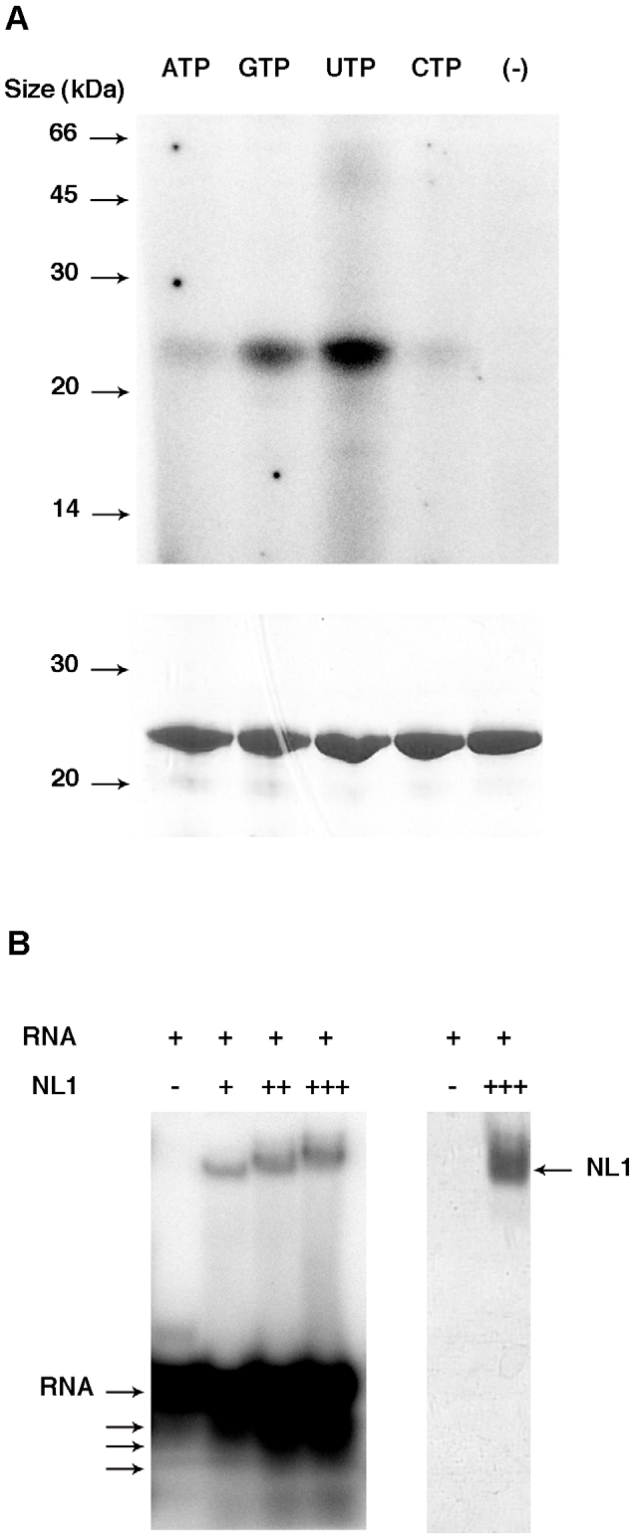
Nucleotide and RNA binding assays of LCMV NL1 domain. **A**, Cross-linking assay. 7 µg of purified protein were incubated in the absence (−) or presence of each indicated radiolabelled NTP. The mixture was then UV-irradiated and loaded onto a denaturing polyacrylamide gel. The latter was analyzed by autoradiography (top) and Coomassie blue staining (bottom). **B**, Band shift assay. Radiolabelled RNA was incubated with increasing quantities (1.4 µg (+), 4.2 µg (++) and 7 µg (+++)) of NL1 protein. Reaction mixture was then analyzed by PAGE, and the gel was visualized by autoradiography (left) and Coomassie blue staining (right). Apparent degradation products are indicated by arrows under the RNA input arrow.

Recent crystal structures of complexes of PA_N_ with three different nucleoside monophosphates show that PA_N_ binds nucleotides [26]. The ability of NL1 to bind nucleotides was investigated using UV-crosslink experiments. We observe that NL1 binds NTPs, preferably UTP and GTP, whereas ATP and CTP show a weaker association (Figure 3A). The PA_N_ structures were determined in complex with ATP, CTP and UTP but not GTP [26] whereas NL1 bind GTP in a stronger fashion than ATP or CTP. The crystal structure relatedness to the endonuclease fold would suggest that the NL1 domain is able to bind RNA rather than nucleotides. We tested RNA binding by NL1, and found that indeed, NL1 binds RNA (Figure 3B). The band shift assay is also suggestive that the RNA substrate is cleaved under the assay conditions, as judged by degradation products at the bottom of the gel under the labeled RNA oligo (Figure 3B). Therefore, we surmise that nucleotide binding properties observed here reflect the ability of NL1 to bind RNA with some sequence specificity in the cap-snatching pathway (see below).

**Figure 4:**
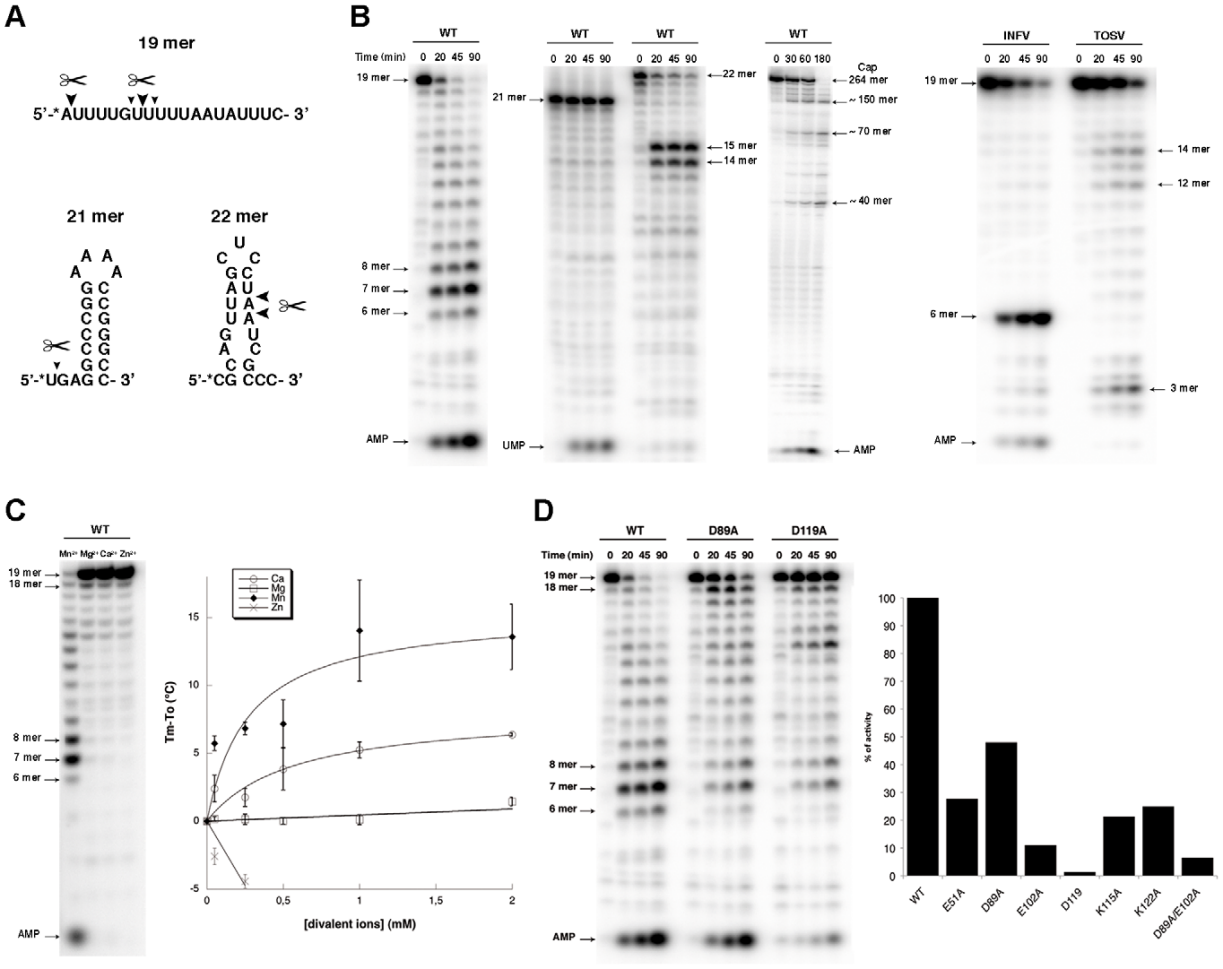
Endonuclease activity of NL1. **A**, Nucleotide sequence of the radiolabelled RNA used for the activity assays. The * indicates the radiolabelled nucleotide. Big and small triangles indicates the primary and secondary cleavage site by wild-type (WT) NL1, respectively. **B**, Kinetics of endonuclease activity of WT NL1 on different substrates (left), and for Influenza and Toscana virus (INFV, TOSV) endonuclease domain (right). Activity assays were performed as described in Materials and Methods, using 3.3 µM of RNA and 1 µM of protein. Reactions were quenched by the addition of EDTA/formamide, and analyzed using 20% polyacrylamide/7M urea gel. Substrate and degradation product sizes are indicated. **C**, Divalent cations effect on the NL1 activity. The reaction was allowed to proceed during 45 min as described in Materials and Methods. The divalent cation assay (left) was run during 45 min without intermediate points. Titration of divalent ions on NL1 by thermal shift assay (right). T_m_ is the melting temperature of NL1 with the divalent ions ; T_o_ is the melting temperature of the protein alone. **D**, Mutational analysis of NL1 domain on the endonuclease activity. Kinetics were performed as described above, with WT, D89A and D119A mutants (Left). Graph showing the % of endonuclease activity determined using FujiImager normalized quantitation for the different mutants (right).

Several synthetic RNA oligonucleotides were used to characterize the endonuclease activity (Figure 4). NL1 is able to cleave ssRNA having no stable secondary structure at specific sites indicating a preference for the presence of uracil (Figure 4A, B), and adenosine to a lesser extent. Likewise, a moderately stable RNA hairpin containing uracil (ΔG = −3.4 kcal/mole) is cleaved down to a 14/15-mer product whereas a stable (ΔG = −14.7 kcal/mole) RNA hairpin devoid of uracil remains unattacked even in its single stranded regions (Figure 4A, B). PolyU RNA is cleaved randomly down to a 8-mer product with a better efficiency than polyA, whereas polyC is not a substrate for NL1 (not shown). A 5′-terminal nucleoside uracil or adenosine 5′-monophosphate is also cleaved and the 5′-monophosphate RNA end apparently competes for internal cleavage. A 5′-capped RNA of 264 nucleotides in length also acts as a substrate. It is cleaved at several specific positions indicated by the sequential appearance of band products over time (Figure 4B). This indicates that the cap structure does not seem to be a direct RNA binding determinant. A *Phlebovirus* (Toscana) virus endonuclease domain was prepared according to bio-informatic predictions described above. Its endonuclease activity was compared to both that of arenavirus NL1 and the influenza H5N1 endonuclease [16]. The enzymes were equally active using short RNA substrates, although it is apparent that sequence-specific cleavage is different for each enzyme: the influenza enzymes prefers cleavage at puric sites, Toscana virus and LCMV enzymes prefer adenosine- and uracil-containing sites (Figure 4B). NL1 is ∼90-fold more active in the presence of Mn^2+^ than Mg^2+^, and shows background activity with Ca^2+^ and Zn^2+^(Figure 4C and not shown). The Mn^2+^ ion has also a significant stabilizing effect as judged by thermostability studies, whereas Zn^2+^ has a deleterious effect.

Mutagenesis analysis of most residues identified as part of the active site (Figure 2A) impaired the endonuclease activity. The most drastic effect was observed for D119, but residual activity was scored for E51, D89, and less for E102 (Figure 4D). As these three residues might coordinate metal ions as proposed above, defective metal-binding due to a point mutation might be compensated by the presence of the remaining two adjacent acidic residues. A double mutant D89A/E102A shows further reduced but not abolished activity. Mutations K115A and K122A generated strongly altered activity, but the similar level of residual activity does not allow the identification of which lysine is predominant in catalysis.

### The Endonuclease Activity is Essential for RNA Transcription, not Replication

**Figure 5:**
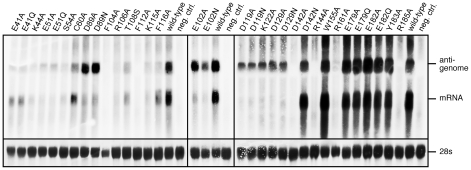
Mutational analysis of the L protein in the context of the LCMV replicon system. Synthesis of antigenomic RNA and Renilla luciferase mRNA was analyzed by Northern blotting. Negative control cells (neg. ctrl.) expressed mini-genome, NP, and an L protein mutant with a mutation in the catalytic site of the RNA-dependent RNA polymerase. The methylene blue-stained 28S rRNA is shown below the blots as a marker for gel loading and RNA transfer. Each panel represents an independent experiment with separate controls. Careful examination of the blots revealed residual signals at the mRNA position for some mutants negative in Renilla luciferase assay. Thus, these signals do not correspond to functional mRNA, but may be prematurely terminated antigenome.

The effect of 33 mutations in L1 on virus RNA and protein expression was studied in a cell-based mini-replicon system. The LCMV L protein mediates the synthesis of two RNA species: first, capped mRNA terminating within the intergenic region, and second, antigenomic RNA being a full-length copy of the genomic RNA template [9], [27]. This dual role in RNA synthesis is recapitulated in the mini-replicon system. It contains all trans-acting factors (L protein and NP) required for transcription and replication of a genome analogue containing Renilla luciferase as a reporter gene (mini-genome). Reporter gene expression was measured in luciferase assay (Table 2), while RNA synthesis was measured in Northern blot (Figure 5), in which luciferase mRNA and antigenome can easily be distinguished due to their size difference. Wild-type (WT) L protein led to expression of high levels of Renilla luciferase (2–3 log units signal-to-noise ratio) as well as Renilla luciferase mRNA and antigenome in a ratio of about 1∶1. Expression of mutant L protein was verified by immunoblotting (Figure S3).

**Table 2:** Functional Analysis of L Protein Mutants in LCMV Mini-Replicon System.

Mutant^1^	Renilla luciferase activity (sRLU)		RNA expression level (Northern blot signal)	
	% of wild-type^2^	Signal-to-noise ratio^3^	Antigenome level, % of wild-type^4^	mRNA-to-antigenome ratio, relative to wild-type^5^
E41A	44.3	243.2	21.9	2.01
E41Q	42.4	310.7	25.5	1.95
K44A	30.2	215.8	23.6	0.93
**E51A**	**1.3**	**8.5**	**28.5**	**0.39**
**E51Q**	**0.5**	**3.6**	**32.9**	**0.26**
S54A	23.5	175.6	28.3	0.80
C60A	47.9	357.7	62.4	1.13
**D89A**	**0.5**	**3.5**	**107.6**	**0.20**
**D89N**	**0.5**	**3.8**	**138.5**	**0.19**
**E102A**	**0.9**	**3.5**	**119.3**	**0.22**
**E102N**	**3.3**	**13.6**	**78.2**	**0.26**
F104A	0.6	3.8	—	—
R106A	4.4	32.5	—	—
T108S	29.7	212.7	48.0	0.89
F112A	0.3	1.8	—	—
K115A	0.9	6.8	—	—
F116A	27.4	209.4	39.1	1.79
**D119A**	**2.0**	**12.5**	**47.5**	**0.06**
**D119N**	**8.0**	**43.0**	**46.4**	**0.07**
**K122A**	**1.6**	**9.3**	**55.3**	**0.04**
**D129A**	**1.3**	**6.5**	**56.5**	**0.04**
**D129N**	**1.3**	**7.4**	**54.1**	**0.05**
D142A	2.8	17.6	—	—
D142N	42.8	270.6	66.1	0.93
R144A	2.3	10.2	—	—
W155A	34.7	203.3	91.0	0.91
R161A	3.4	17.6	—	—
E179A	40.0	239.4	109.9	0.46
E179Q	25.4	174.6	105.0	0.44
E182A	32.6	201.3	154.9	0.40
E182Q	29.3	184.9	103.1	0.42
Y183A	16.9	98.8	82.3	0.48
R185A	0.3	1.4	—	—

1 Mutants with selective defect in mRNA synthesis are shown in boldface.

2 Standardized relative light unit (sRLU) value (wild-type = 100%). Mean of ≥2 independent transfection experiments.

3 sRLU value of mutant divided by sRLU value of negative control mutant containing a mutation in the catalytic site of the RNA-dependent RNA polymerase. Mean of ≥2 independent transfection experiments.

4 Antigenome signals in Northern blots were quantified via intensity profiles (wild-type = 100%).

5 RNA signals in Northern blots were quantified and the mRNA-to-antigenome signal ratio was calculated. The wild-type ratio was set at 1 for each experiment (i.e. the signal ratio of a mutant was normalized with the wild-type ratio) to render independent blots comparable.

The phenotype of mutants E41A, E41Q, K44A, S54A, C60A, T108S, F116A, D142N, and W155A is similar to that of wild-type. Mutants E179A, E179Q, E182A, E182Q, and Y183A also express luciferase and RNA at high level, but the steady-state level of mRNA relative to that of antigenome is reduced by about 50%. Mutants F104A, R106A, F112A, K115A, D142A, R144A, R161A, R185A neither express Renilla luciferase nor any RNA species, indicating that global functions of L protein are affected.

The most interesting phenotype is observed with mutants D89A, D89N, E102A, E102N, D119A, D119N, K122A, D129A, and D129N. They synthesize antigenome close or equal to wild-type level, but are defective in mRNA and, thus, reporter gene expression (Figure 5 and Table 2, shown in boldface). A similar phenotype is seen with mutants E51A and E51Q, though associated with reduced antigenome level. These data indicate that residues E51, D89, E102, D119, K122, and D129A are essential for viral mRNA synthesis, but not required for expression of uncapped RNA species. With the exception of the D129 residue located at the surface of the protein remote from the endonuclease active site, it is remarkable that these transcription-null mutants form the catalytic site (Figure 2) and match precisely those of the PD…(D/E)XK endonuclease type II signature sequence.

## Discussion

The structural and functional results presented here show that the LCMV NL1 domain is an RNA endonuclease. The uncoupling of RNA replication from transcription and selective disappearance of mRNA when NL1 active site residues are mutated strongly suggests that this activity is involved in cap-snatching.

The identification of the arenavirus endonuclease is in line with the recent discovery of the PA_N_ endonuclease domain of influenza virus. Whereas the active site of influenza virus features a cluster of three acidic residues, the active site of arenavirus contains four acidic residues (E51, D89, E102 and D119), as well as two important lysine residues K115 and K122 neighboring D119 (Figure 2A). The NL1 active site resembles but is clearly distinct from that of influenza PA_N_. Indeed, there is no histidine in the catalytic center, and the arenavirus NL1 nuclease has some specific features both upstream and downstream of the PD signature sequence. We define the arenavirus endonuclease motif as E-X_38_-P-D-X_(11,13)_-E-X_12_-K-X_3_-D-X_2_-K. The most obvious difference with the only known related RNA endonuclease, that of influenza virus PA_N_, is a divergence upstream the PD motif in structural elements carrying the E51 residue (Figure 1C), and the presence of a triad K…D…K at the distal side of the latter signature sequence (Figure 2A). Contrary to PA_N_ which shares a conserved and essential histidine involved in the binding of both the metal ion and a nucleotide onto helix α3 [15], [16], [26], NL1 does not possess this conserved histidine residue. Instead, NL1 has a glutamic acid residue E51, which might reflect a different nucleobase specificity as detected in our nuclease assays (Figure 4). Likewise, residues downstream the PD motifs are distinct from the consensus sequence, and differently organized into a triad including two lysines. The presence of water molecules and previous structural models for influenza PA_N_ allows to propose putative positions of metal ions, coordinated by D89 and E102.

The first step in the general mechanism for phosphodiester hydrolysis is the preparation of the attacking nucleophile by deprotonation, usually involving a general base deprotonating a water molecule. Lysine is often considered as this general base candidate in endonucleases but is not strictly conserved [28], [29]. Here, there are no indications against D119 being this general base. Alternately, it could well be either lysine K115 or K122. Both are oriented towards the active site, and they could well have their pKa lowered by D119 in order to initiate the reaction. Reverse genetic studies provide evidence for K122, not K115. Indeed, mRNA production is selectively abolished and clearly uncoupled from RNA synthesis in the case of K122A mutant, while the K115A mutant was completely defective preventing interpretation of its role in the endonuclease catalytic site. Although it is not known if uncapped mRNAs are synthesized and degraded for the transcription-null mutants, the most plausible scenario is that primer shortage prevents significant capped mRNA synthesis. Overall, the replicon data presented here closely match those obtained on the closely related Lassa arenavirus using a similar replicon system [30]. Arenaviruses may thus use two clearly independent and distinct RNA synthesis priming mechanisms: one is dependent on an active endonuclease carried by the N-terminus of the L protein, and the other might be linked to the observation that an extra G residue is found at the 5′-end of arenavirus genomes and antigenomes. The latter G bases would thus reflect a yet-uncharacterized priming mechanism unrelated to the U/A cleavage sequence preference of NL1.

NL1 also binds nucleotides, but the NTP binding site should differ from that of PA_N_. Indeed, the influenza PA_N_ histidine 41 is involved in binding the nucleobase of the presumed incoming RNA substrate. The NL1 endonuclease does not share the same sequence specificity, and E51 is positioned at a spatially equivalent position.

The cap structure does not seem to be a direct RNA binding determinant (Figure 4B), as endonucleolytic cleavage is not directed to cleavage sites preferentially in the vicinity of the cap. We thus infer that an independent cap-binding site way exist elsewhere in viral proteins to bind and select cellular mRNAs, a possibility reminiscent of influenza for which PA carries the endonuclease activity and PB2 the cap binding site [15], [16], [31].

Structure and sequence alignment studies show that the N-terminal endonuclease domain of the L protein is also conserved in the *Bunyaviridae* family, although the *Nairovirus* endonuclease domain is not located into the N-terminal end of the protein. These findings were confirmed by the endonuclease activity of the N-terminal end of the L protein of TOSV (Figure 4B). Thus, we provide evidence that all three segmented negative single-strand RNA virus species share an endonuclease domain probably involved in the cap-snatching process during the viral life cycle. These data raise the question of a possible common ancestor for these viruses. Indeed, these three virus families use a cap-snatching mechanism involving binding and cleavage of cellular mRNA caps subsequently used by a large primer-dependent RNA-dependent RNA polymerase. It seems more plausible that the L gene has evolved by divergence over time, rather than by multiple acquisitions of several activities converging into a common structure, at least in the case of the endonuclease. Furthermore, our study raises the interesting possibility that other activities involved in RNA replication/transcrition might be discovered by comparative analysis of *Orthomyxoviridae* PB1, PB2, PA and *Arenaviridae/Bunyaviridae* L proteins.

To our knowledge, a single crystal structure of a functional arenavirus protein is currently available, that of the Machupo virus glycoprotein GP1 in complex with its human receptor, TfR1 [32]. Our results provide an arenavirus L domain structure, with a role consistent with the hypothesis of a cap-snatching mechanism suggested for arenaviruses [9], [10]. The strategy used here to produce individually active domains might be useful to further characterize the *Arenaviridae/Bunyaviridae* large L protein which had so far resisted all biochemical characterization attempts.

The influenza, *Arenaviridae* and *Bunyaviridae* endonucleases are so far the only three examples of RNA endonucleases similar to type II DNA restriction endonucleases. The presence of such an endonuclease suggests that it could serve as a fruitful target for antiviral strategies against these two families, since such kind of inhibitors have been reported in the case of the influenza virus [33], [34], [35].

## Materials and Methods

### Cloning, Expression and Purification of LCMV NL1 Domain

The LCMV NL1 cDNA (Armstrong strain, aa 1 to 196) was cloned into pDest14 with a N-terminus hexa-histidine tag and expressed in *E.coli* Rosetta (DE3) pLysS (Novagen), at 17°C in 2YT medium overnight after induction with 500 µM IPTG. Cell pellets from harvested cultures were resuspended in 50 mM Tris buffer, pH 8.0, 300 mM NaCl, 10 mM imidazole, 0.1% Triton, 5% Glycerol. Lysozyme (0.25 mg/ml), PMSF (1 mM), DNase I (2 µg/ml), and EDTA free protease cocktail (Roche) were added before sonication. IMAC chromatography of clarified lysates was performed on a 5 ml His prep column (Akta Xpress FPLC system, GE Healthcare) eluted with imidazole. Size exclusion chromatography was performed on preparative Superdex 200 column (GE Healthcare) pre-equilibrated in 10 mM Imidazole, pH 8.0, 50 mM NaCl, 2 mM DTT. Protein was concentrated (28 mg/ml) using a centrifugal concentrator. For enzymatic studies, WT and mutants were express in the *E.coli* BL21 star strain (Invitrogen) and further purified on HiTrap Q sepharose 1 ml column (GE Healthcare) to remove *E. coli* RNase contaminants. Proteins eluted in a linear gradient from 50 mM to 1 M NaCl in 10 mM Hepes buffer, pH 7.5, 2 mM DTT. A synthetic gene of the H5N1 PA_N_ endonuclease was designed as described [16]. The Toscana virus (strain France AR_2005, aa 2 to 233) cDNA was obtained from infected cell cultures. Both ORFs were cloned as a N-terminal Thioredoxin-Hexahistidine fusion in pETG20A. The tag was cleaved using TEV protease before a final gel filtration.

### Crystallization

Crystals grew in LiSO_4_ 250 mM, citrate 50 mM, isopropanol 5.5%, using the hanging drop vapor diffusion method in Linbro plates by mixing 1 µl of protein solution with 1 µl of reservoir solution. Crystals were cryoprotected by dipping in a solution containing 65% of crystallization buffer and 35% of a buffer made of size exclusion chromatography buffer/glycerol (50/50). Crystals were cryo-cooled in liquid N_2_. The crystals belong to space group C222_1_ and have two molecules per asymmetric unit. Despite repeated attempts, crystal soaked into the above buffer supplemented with various concentrations of MnCl_2_ yielded crystals diffracting to >4 Å.

### Data Collection and Structure Determination

Diffraction intensities were recorded on the ID14-4 beamline at the European Synchrotron Radiation facility, Grenoble, France. Data were processed and integrated with MOSFLM [36]. Scaling and merging of the intensities was performed with SCALA and programs from the Collaborative Computational Project, No. 4 (CCP4) suite [37]. The structure was determined using SAD data from one selenomethionylated protein crystal diffracting to 3.4 Å resolution with SHARP/autoSHARP, followed by density modification with SOLOMON and DM. An initial model was built using BUCCANEER and completed in COOT, followed by refinement using BUSTER (see Text S1). Details of structure determination are given as supplemental material. Data from a native crystals diffracting to a 2.13-Å resolution were collected on an ADSC QUANTUM 315r at a wavelength of 0.9835 Å. The structure was refined with BUSTER and COOT using this data set (Table 1) [38]. The atomic coordinates have been deposited at the PDB (3JSB).

### Sequence Retrieval

A PHI-BLAST search using the sequence corresponding to the L1 domain and the signature of the *Arenaviridae* endonuclease motif *i.e.* P-D-_x(11,13)_-E-_x(12)_-K-_x(3)_-D-_x(2)_-K ; was performed against non-redundant databases [39]. After 3 iterations, Batai and Kairi viruses both belonging to *Orthomyxoviridae*, appears in the section with an E-Value below threshold. A fourth iteration including these two sequences allows retrieving the entire family of orthomyxoviruses, with E-value comprised between 3e^−18^ and 2e^−4^.

A standard CDD search from the sequence of Tensaw virus allows retrieving all the L of the *Bunyaviridae* family hitting the pfam 04196 [40].

### Sequence Comparison

A multiple sequence alignment of the N-terminal end of the L protein from LCMV, HLCV, BUNV, HANV, PUMV, RVFV, TOSV, TOMV, WTMV, CCGV, DUGV, was first performed with the T-coffee algorithm (http://tcoffee.vital-it.ch/cgi-bin/Tcoffee/tcoffee_cgi/index.cgi). Using the secondary structure prediction of the endonuclease domain of L proteins, the putative conserved active site residues were identified and placed correctly in the alignment.

### UV-Crosslink Experiments

7 µg of purified protein were incubated for 15 min at 25°C, with 0.5 µl of the various α-^32^P NTP (0.4 µCi/µl) in 10 µl of reaction buffer containing 10 mM Imidazole, pH 8.0, 50 mM NaCl, 2 mM DTT. The reaction mixtures were then exposed to UV light (254 nm) for 6 min at 5 mm distance. The crosslinked species were separated in a 15% polyacrylamide denaturing gel and visualized by autoradiography using photo-stimulated plates and a Fujilmager (Fuji).

### RNA Binding Experiments

The RNA 5′-AUUUUGUUUUUAAUAUUUC-3′ (Ambion) was [^32^P] 5′-end labeled, and 0.4 µM of radiolabelled RNA was incubated 20 min at 25°C without and with 1.4 µg, 4.2 µg and 7 µg of protein in 10 µl of 10 mM Imidazole, pH 8.0, 50 mM NaCl, 2 mM DTT. Reaction mixtures was analyzed by PAGE and visualized by autoradiography.

### Ion Binding Assays

Titration curves with CaCl_2_, MnCl_2_, MgCl_2_ and ZnCl_2_ were performed at 1 mg/ml protein in gel filtration buffer using thermal shift assay. Technical details can be be found in [41].

### Endonuclease Assays

Endonuclease activity was assayed using 4 different heteromeric RNA substrates: an unstructured 19 mer as described above, a 21 mer stable hairpin (5′-UGAGGCCCGGAAACCGGGGCC-3′ (Ambion), ΔG = −14.7 Kcal/mole), a 22 mer moderately stable hairpin (5′- CGCAGUUAGCUCCUAAUCGCCC-3′ (Ambion), ΔG = −3.4 Kcal/mole), and a long 264 mer RNA corresponding to the SARS-CoV 5′-genome sequence. The latter was radiolabelled with a cap structure at its 5′-end using the ScriptCap m7G Capping System (Epicentre *Biotechnologies*) with [α^32^P]GTP. Endonuclease assays were carried out using 3.3 µM of radio-labeled RNA in a buffer containing 40 mM Tris-base, pH 7.5, 100 mM NaCl, 10 mM β-Mercaptoethanol and 2 mM MnCl_2_. Reactions were initiated by the addition of 1 µM of protein and incubated at 37°C, and stopped by the addition of EDTA/formamide. Reactions products were analyzed using denaturing polyacrylamide gel electrophoresis (20% polyacrylamide, 7 M urea in TTE buffer (89 mM Tris, 28 mM taurine, 0.5 mM EDTA) and analyzed by autoradiography.

### Mutagenesis and Reverse Genetics Assays Using a LCMV Mini-Replicon System

The LCMV replicon system is based on strain Armstrong clone 13 and has been established in analogy to the Lassa virus replicon described previously [42]. BSR T7/5 cells constitutively expressing T7 RNA polymerase [43] were transiently transfected with T7 promoter-driven expression constructs for L protein, nucleoprotein (NP), mini-genome (MG) containing Renilla luciferase reporter gene, and firefly luciferase as a transfection control. L protein mutants were generated as described [44]. One day after transfection, total RNA was prepared for Northern blotting and cell lysate was assayed for firefly and Renilla luciferase activity. Renilla luciferase levels were normalised with firefly luciferase levels resulting in standardized relative light units (sRLU). Northern blot was performed using an antisense ^32^P-labeled riboprobe targeting the Renilla luciferase gene. Autoradiography was quantified on a PhosphorImager (Amersham Biosciences). To verify protein expression, hemagglutinin (HA)-tagged L protein was expressed in BSR T7/5 cells inoculated with modified vaccinia virus Ankara expressing T7 RNA polymerase (MVA- T7) [45] and detected in immunoblot using anti-HA antibody.

## Supporting Information

Figure S1 Sequence Alignment of Viral Endonuclease Domains. **A**, Structure-based sequence alignment of NL1 with the two influenza PAN (3EBJ, 2W69), Tt1808 (1WDJ) and SdaI (2IXS), showing the structurally-conserved endonuclease motif (Highlighted in red). **B**, Sequence alignment of arenavirus NL1: Lymphocytic choriomeningitis virus (LCMV), Dandenong virus (DANV), Mopeia virus (MOPV), Morogoro virus (MORV), Mobala virus (MOBV), Ippy virus (IPPV), Lassa virus (LASV), Lujo Virus (LUJV), Parana virus (PARV), Pichinde virus (PICV), Allpahuayo virus (ALLV), Chapare virus (CHAV), Tamiami virus (TAMV), Whitewater Arroyo virus (WWAV), Bear Canyon virus (BCNV), Flexal virus (FLEV), Pirital virus (PIRV), Amapari virus (AMAV), Guanarito virus (GTOV), Cupixi virus (CPXV), Machupo virus (MACV), Junin virus (JUNV), Tacaribe virus (TCRV), Sabia virus (SABV), Oliveros virus (OLVV), Latino virus (LATV). Residues in a solid red background are strictly conserved. The blue point indicates the key active site residues.
(4.29 MB TIF)

Figure S2 Conservation of NL1 Between Arenaviruses and Bunyaviruses. **A**, Sequence alignment showing endonuclease motif (highlighted in red) of the NL1 domain from LCMV and the five genus of the *Bunyaviridae* families. Each genera is represented by two viruses: *Orthobunyavirus*: Human La Cross Virus (HLCV) and Bunyamwera virus (BUNV), *Hantavirus*: Hantaan virus (HANV) and Puumala virus (PUMV), *Phlebovirus*: Rift valley fever virus (RVFV) and Toscana virus (TOSV), *Tospovirus*: Tomato virus (TOMV) and Watermelon silver mottle virus (WTMV), *Nairovirus*: Crimean-Congo hemorrhagic fever virus (CCGV) and Dugbe virus (DUGV). Position of the start of the motif is labelled in blue for each virus. **B**, Based on secondary structure predictions topology diagrams were drawn for the NL1 domains from PUMV, RVFV, TOMV, HANV, CCGV. Colours are the same as in Figure 1D. N-terminal and C-terminal position are labelled with the aa position. The two red dots, the black dot and the green dot indicate the PD, E/D, and K residues, respectively, from the key active site. Principal conserved secondary structures are labelled as for LCMV NL1 domain.
(8.97 MB TIF)

Figure S3 Verification of L protein expression by immunoblotting. L protein used for analysis in replicon assay was tagged with HA tag, expressed under T7 promoter control in cells inoculated with modified vaccinia virus Ankara expressing T7 RNA polymerase (MVA-T7), and detected in immunoblot using anti-HA antibody. MVA, cells inoculated with MVA-T7 but not transfected; neg. ctrl., L mutant containing a mutation in the catalytic site of the RdRp.
(1.13 MB TIF)

Text S1 Supplementary Methods.
(0.08 MB DOC)
