# Supplementary figures and images for: The N-Terminal Domain of the Arenavirus L Protein Is an RNA Endonuclease Essential in mRNA Transcription
Source: PLoS Pathog. 2010 Sep 16;6(9):e1001038. doi: 10.1371/journal.ppat.1001038 (PMC2940758; doi:10.1371/journal.ppat.1001038)

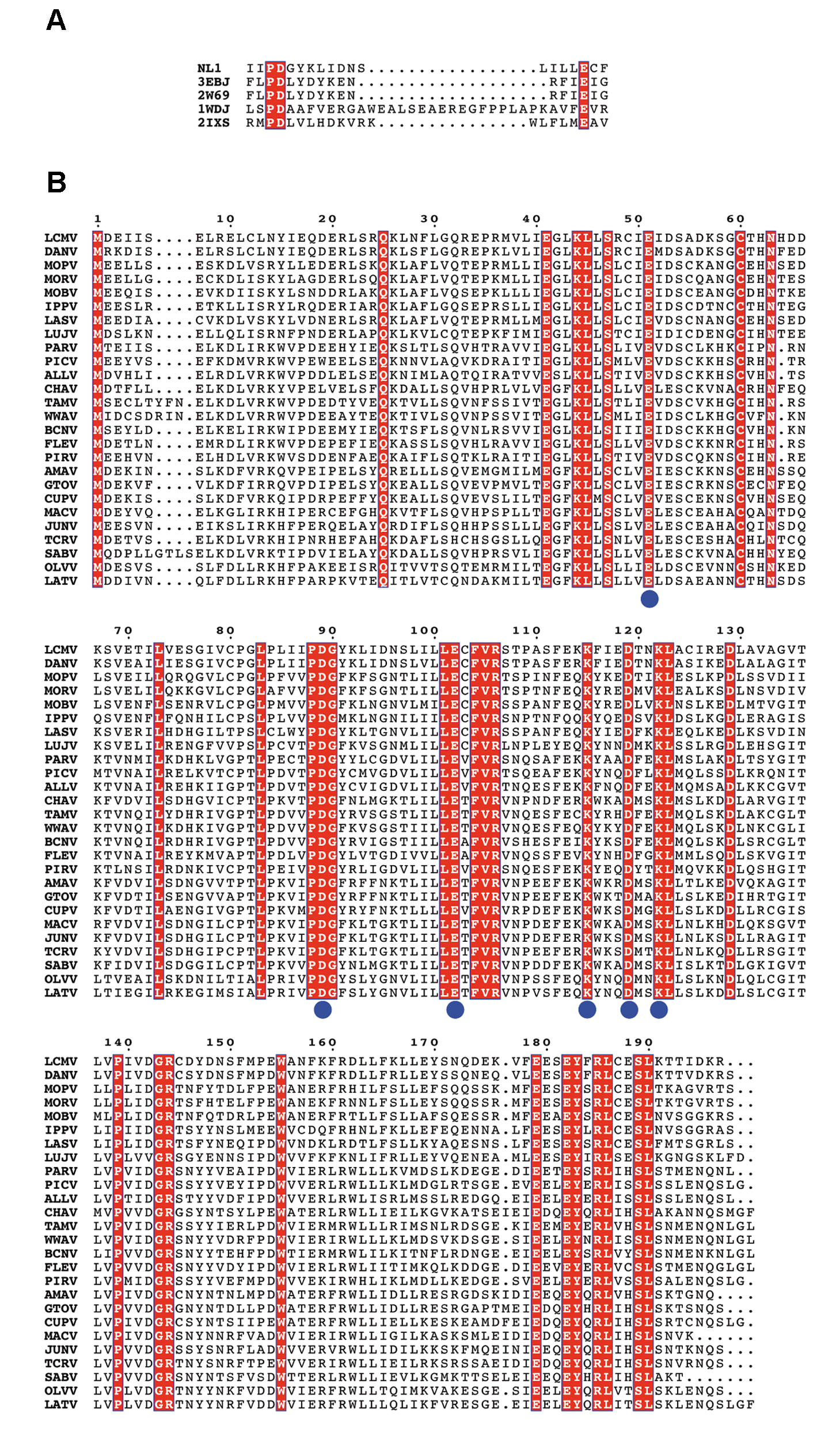

Supplement: Figure S1 — Sequence Alignment of Viral Endonuclease Domains. A, Structure-based sequence alignment of NL1 with the two influenza PAN (3EBJ, 2W69), Tt1808 (1WDJ) and SdaI (2IXS), showing the structurally-conserved endonuclease motif (Highlighted in red). B, Sequence alignment of arenavirus NL1: Lymphocytic choriomeningitis virus (LCMV), Dandenong virus (DANV), Mopeia virus (MOPV), Morogoro virus (MORV), Mobala virus (MOBV), Ippy virus (IPPV), Lassa virus (LASV), Lujo Virus (LUJV), Parana virus (PARV), Pichinde virus (PICV), Allpahuayo virus (ALLV), Chapare virus (CHAV), Tamiami virus (TAMV), Whitewater Arroyo virus (WWAV), Bear Canyon virus (BCNV), Flexal virus (FLEV), Pirital virus (PIRV), Amapari virus (AMAV), Guanarito virus (GTOV), Cupixi virus (CPXV), Machupo virus (MACV), Junin virus (JUNV), Tacaribe virus (TCRV), Sabia virus (SABV), Oliveros virus (OLVV), Latino virus (LATV). Residues in a solid red background are strictly conserved. The blue point indicates the key active site residues. (4.29 MB TIF) [file ppat.1001038.s001.tif]

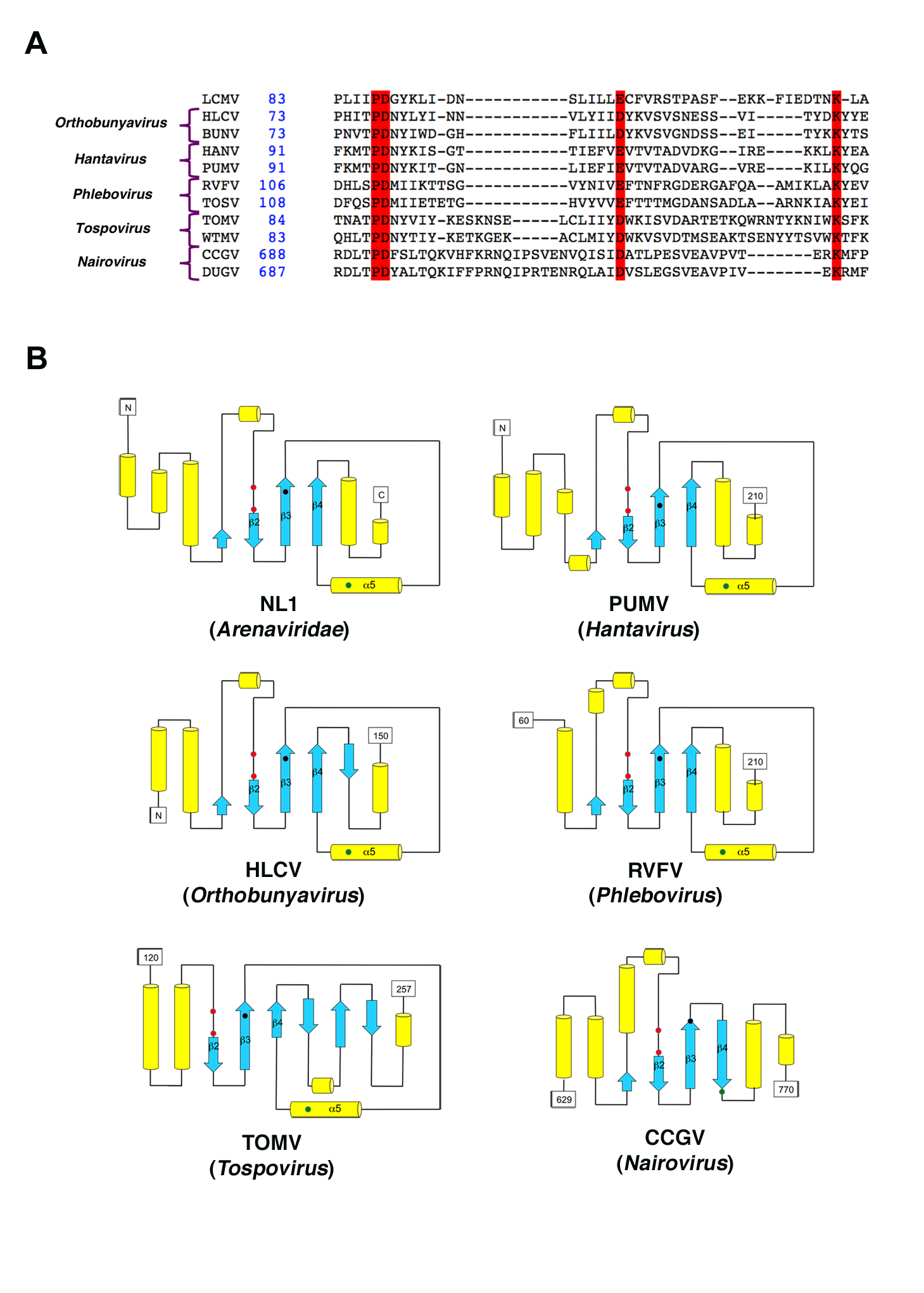

Supplement: Figure S2 — Conservation of NL1 Between Arenaviruses and Bunyaviruses. A, Sequence alignment showing endonuclease motif (highlighted in red) of the NL1 domain from LCMV and the five genus of the Bunyaviridae families. Each genera is represented by two viruses: Orthobunyavirus: Human La Cross Virus (HLCV) and Bunyamwera virus (BUNV), Hantavirus: Hantaan virus (HANV) and Puumala virus (PUMV), Phlebovirus: Rift valley fever virus (RVFV) and Toscana virus (TOSV), Tospovirus: Tomato virus (TOMV) and Watermelon silver mottle virus (WTMV), Nairovirus: Crimean-Congo hemorrhagic fever virus (CCGV) and Dugbe virus (DUGV). Position of the start of the motif is labelled in blue for each virus. B, Based on secondary structure predictions topology diagrams were drawn for the NL1 domains from PUMV, RVFV, TOMV, HANV, CCGV. Colours are the same as in Figure 1D. N-terminal and C-terminal position are labelled with the aa position. The two red dots, the black dot and the green dot indicate the PD, E/D, and K residues, respectively, from the key active site. Principal conserved secondary structures are labelled as for LCMV NL1 domain. (8.97 MB TIF) [file ppat.1001038.s002.tif]

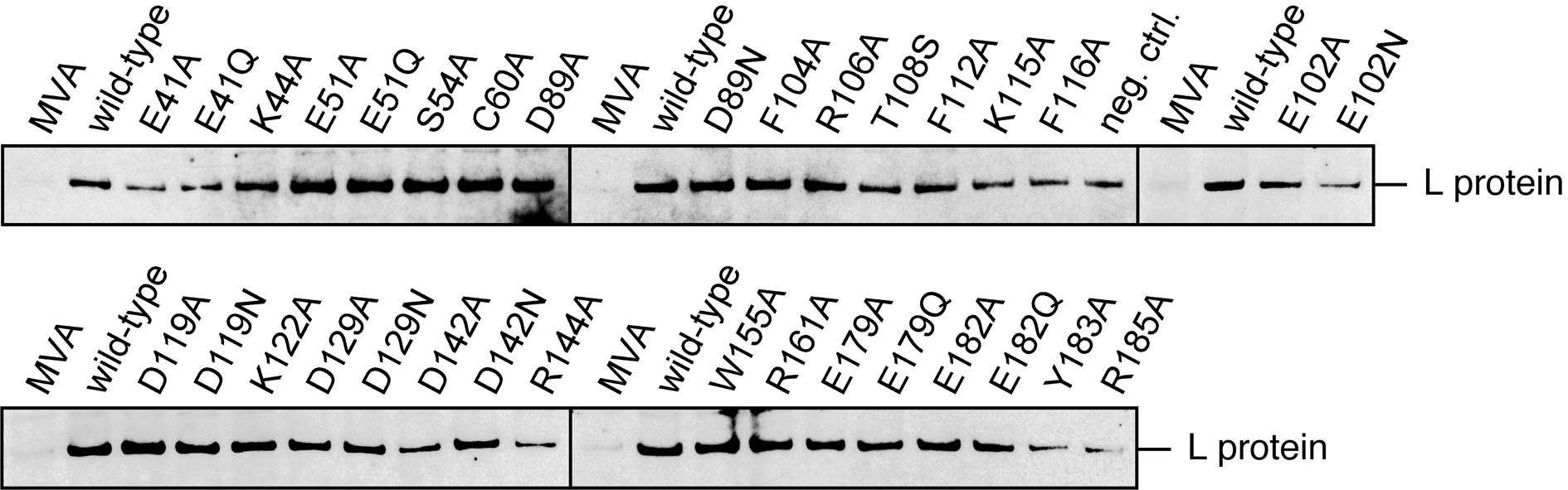

Supplement: Figure S3 — Verification of L protein expression by immunoblotting. L protein used for analysis in replicon assay was tagged with HA tag, expressed under T7 promoter control in cells inoculated with modified vaccinia virus Ankara expressing T7 RNA polymerase (MVA-T7), and detected in immunoblot using anti-HA antibody. MVA, cells inoculated with MVA-T7 but not transfected; neg. ctrl., L mutant containing a mutation in the catalytic site of the RdRp. (1.13 MB TIF) [file ppat.1001038.s003.tif]
