# Supplementary material for: The N-Terminal Domain of the Arenavirus L Protein Is an RNA Endonuclease Essential in mRNA Transcription
Source: PLoS Pathog. 2010 Sep 16;6(9):e1001038. doi: 10.1371/journal.ppat.1001038 (PMC2940758; doi:10.1371/journal.ppat.1001038)
Supplement: Text S1 — Supplementary Methods. (0.08 MB DOC) [file ppat.1001038.s004.doc]

**Supplementary Methods:**

**Data Collection and Structure Determination**

Diffraction intensities were recorded on the ID14-4 beamline at the European Synchrotron Radiation facility using an ADSC QUANTUM 315r detector. An initial Se-MET peak dataset (wavelength = 0.9788 Å) was collected to 3.4 Å resolution and processed with XDS [46] and SCALA [47]. This was used in autoSHARP [48] to find 4 Se sites using SHELXD [49] for the 2 molecules in the asymmetric unit.

Subsequent phasing and refinement in SHARP [50] gave an overall anomalous phasing power of only 0.41 (with the value dropping below one at about 7.0 Å). The resulting phases were hardly good enough to distinguish the correct hand. However, using PROFESSS [37] it was possible to determine the non-crystallographic symmetry operators for the dimer (related by a 2-fold rotation). These could then successfully be used in DM [51] to perform density modification including solvent flattening, histogram matching and NCS averaging.

An iterative protocol was then being used, to first automatically build the model into the current best map using BUCCANEER [52], then feed this partial model into density modification using SOLOMON [53] as implemented in autoSHARP and finally perform NCS averaging in DM. This procedure was repeated with different restraints on the type of structure BUCCANEER should built: either only helical or only beta-sheet structures. The final collection of BUCCANEER models were pooled together to create an initial model in Coot [54] which consisted mainly of poly-ALA and some initial sequence assignments, scattered over several fragments and chains.

This model was refined in BUSTER [55] against the Se-MET peak dataset to a final R/Rfree value of 0.335/0.364. based on that map and model, the dimer could be generated by keeping the correct sequence information, the NCS operator and some manual re-building. The resulting model of 346 residues with about 2/3 of the sequence assigned was again refined in BUSTER and rebuilt in Coot to a final R/Rfree of 0.231/0.278.

A native data set to 2.0 Å resolution was collected at a wavelength of 0.9835 Å and used for the final refinement and model building cycles. The data were processed and integrated with MOSFLM [56] and scaling and merging of the intensities were carried out using SCALA from the CCP4 suite [37]. The initial model from BUSTER was used as a starting point for model refinement using REFMAC [57] and model building in Coot. A final round of BUSTER refinement and manual correction improved the model from an R/Rfree of 0.225/0.271 to final values of 0.199/0.222, using a re-processed (XDS and SCALA) native dataset and enforcing the two-fold NCS in the structure by means of Local Structure Similarity Restraints (LSSR) as described by [58].
